# Long-Distance Transport of Finisher Pigs in the Iberian Peninsula: Effects of Season on Thermal and Enthalpy Conditions, Welfare Indicators and Meat pH

**DOI:** 10.3390/ani11082410

**Published:** 2021-08-16

**Authors:** Genaro C. Miranda-de la Lama, Rubén Bermejo-Poza, Nora Formoso-Rafferty, Malcolm Mitchell, Pilar Barreiro, Morris Villarroel

**Affiliations:** 1 Department of Animal Production & Food Science, Agri-Food Institute of Aragon (IA2), University of Zaragoza, Miguel Servet 177, 50013 Zaragoza, Spain; 2 Department of Animal Production, Veterinary School, Complutense University of Madrid, 28036 Madrid, Spain; rbermejop89@gmail.com; 3 Department of Animal Science, ETSIAAB Technical University of Madrid, 28036 Madrid, Spain; nora.formosorafferty@upm.es (N.F.-R.); morris.villarroel@upm.es (M.V.); 4 Animal & Veterinary Sciences, Roslin Institute, Scotland’s Rural College (SRUC), Midlothian EH25 9RG, UK; malcolm.mitchell5@btinternet.com; 5 Department of Agroforestry Engineering, ETSIAAB Technical University of Madrid, 28036 Madrid, Spain; pilar.barreiro@upm.es

**Keywords:** pig welfare, long-distance transport, enthalpy, thermal stress, meat pH

## Abstract

**Simple Summary:**

Long-distance transport in the global swine industry is more the rule than the exception. We tested the impact on the rates of temperature change and air enthalpy on the stress response and muscle pH in pigs subjected to long-distance travel from Spain to Portugal performed in the summer and winter. We found that winter journeys are more adverse for the animals because during the journey, abrupt variations in rates of temperature change and air enthalpy caused a marked physiological stress response and effects on the meat pH after 45 min. These results indicate the need to develop new environmental control strategies that mitigate abrupt temperature changes during travel to attenuate the biological cost of such long-distance transport on the animals.

**Abstract:**

Current legislation in the European Union places limits on live pig transport according to outside temperature, but less is known about the effects of sudden changes in the thermal microenvironment in trailers, particularly during long-distance transport. In this study, we measured the temperature and relative humidity inside livestock vehicles carrying 1920 Spanish finisher pigs (live weight 100 kg and 240 animals per journey) during eight long-distance (>15 h) commercial journeys to slaughter from northern Spain to Portugal in the summer and winter. Here, we report the rate of change in the air temperature (°C × min^−1^) and air enthalpies in the transport vehicle (kg water kg dry air-1). At sticking, blood samples were taken for to measure cortisol, glucose, and creatine kinase (CK) as stress response indicators, and the meat pH after 45 min and the pH after 24 h were also determined. The rate of change in the air temperature and enthalpy was higher inside the livestock vehicle during the winter months and was positively related with higher cortisol and glucose levels and lower pH after 45 min (*p* < 0.05). It is proposed that the rate of temperature change and air enthalpy represent useful integrated indices of thermal stress for pigs during transport.

## 1. Introduction

Pig production in the European Union is increasingly industrialized and specialized [1], with the EU being the second largest pig producer in the world, with 24.1 million tons of pork produced in 2019 [2]. Spain is one of the most important pig producers in Europe and almost a third of national production is exported [3]. To maintain this high level of competitiveness, the pig sector depends on road transport as one of its strategic components in the European pork supply chain [4]. The high demand for pork meat in some member countries has stimulated intracommunity trade involving the long-distance transport of live pigs [5]. Even under favorable conditions, long-haul transport can cause different degrees of stress to animals, ranging from discomfort and aversion to death [6]. Extreme ambient temperatures during long-distance journeys are considered as one of the most important risk factors for dead on arrivals, non-ambulatory animals, skin lesions, and carcass downgrading, especially when microclimate conditions are outside of the optimal thermal comfort zone for pigs [7]. These thermal conditions may be complex and result from the interaction of several factors such as external climatic conditions, heat and water production from the animals, ventilation regimes, distribution and flow rates, and additional external sources of heat and/or moisture [8].

The temperature humidity index (THI) forms the basis for calculating ventilation on farms [9]. The Livestock Weather Safety Index (LWSI) is derived from the THI and is widely applied across species, as described by Eigenberg et al. [10]. The LWSI has been related to mortalities during pig transport in Denmark [11]. Barbosa-Fihlo et al. [12] have compared using enthalpy with the Black Globe and Humidity Index (BGHI) proposed by Curtis [13] in the assessment of thermal comfort in broilers. Enthalpy was deemed the better index and the same authors [14] later applied an Enthalpy Comfort Index (ECI) to poultry during transport, reporting that hostile thermal conditions are well predicted by this index. Enthalpy, a concept that combines temperature and relative humidity, is the heat energy of the air and is the major determinant of the dry and latent heat loss to the environment. It can be calculated using simple tools (i.e., a thermometer and hygrometer) and mathematical models [15]. Advances in this area will underpin improvements in handling and the transport of livestock, especially under more extreme climate conditions.

Current legislation in Europe: European Union Council Regulation 1/2005 [16] defines temperature limits during the transport of livestock on journeys over 8 h in duration. However, the legislation does not specify any corresponding limits for humidity or water vapor content in the air. Similarly, there is no consideration of the effects upon the animals regarding the rate of change of the thermal conditions. According to EC 1/2005, the upper temperature limit for the long-distance transport of livestock on long journeys is 30 °C with a tolerance of 5 °C, meaning the absolute limit is 35 °C. Although integrated indices of temperature and humidity are used extensively in other areas of animal production to predict their impact on production and welfare, this approach has not been applied to animal transport in the European legislative context, and few studies have considered the potential application of enthalpy as an integrated index of thermal load. In this study, the aim was to develop and compare psychrometric charts as well as rates of change in the temperature and enthalpy from long distance (>8 h) journeys carrying pigs across the Iberian Peninsula from northern Spain to southern Portugal. In addition, the potential use of rates of change of temperature and enthalpy as non-invasive indicators and their impact on blood stress indicators and meat pH in pigs were evaluated.

## 2. Materials and Methods

A total of eight long-distance commercial journeys from a pig finishing farm located in the town of Campanas in the autonomous community of Navarra in Spain (42.69 N, 1.65 W, and 575 m.a.s.l.) to a EU licensed abattoir at Vila Franca do Rosario in the Lisbon region in Portugal (38.97 N, 9.25 W, and 88 m.a.s.l.) were studied. The distance for all of the journeys was 936.5 km with an average duration of 14 h and 30 min. Campana’s climate is Cf2b according to Köppen–Geiger’s climate classification; warm and humid with cold winters and mild summers with rainfall throughout the year, except for two relatively dry months. The annual average temperature is around 12 °C with an average monthly temperature of 5 °C in the coldest month (January) and 20.9 °C in the warmest month (August), attending to the typical climate data [17]. Pigs were carefully handled during the pre-slaughter period: they were handled at unloading and in lairage using plastic paddles only. They were loaded and unloaded by the workers hired by the farm and the abattoir and were kept together in familiar groups during transport and lairage. All procedures were conducted in accordance with the guidelines for the ethical treatment of animals in applied animal welfare studies [18].

### 2.1. Study Description

The eight long-distance journeys monitored the transport of a total of 1920 Spanish finisher pigs (240 animals per journey) in the winter (*n* = 3) and in the summer (*n* = 5). All of the pigs were Large White/Landrace × Duroc pigs with a mixed group males and females who were six-months-old (with an approximate average live weight of 100 kg). Pigs were off feed for 12 h before transport. Loading was usually conducted at approximately 05:00 a.m. by three farm operators, avoiding mixing between unfamiliar pens. The farm operators went to one pen at a time to drive the pigs toward the loading platform using plastic bags and plastic boards. The loading procedures lasted approximately 2 h per journey. The animals were loaded and transported from Campanas (Spain) to a commercial abattoir in Vila Franca do Rosario (Portugal). This journey duration is compliant with the overarching transport regulation, EC 1/2005, which prescribes a maximum journey time for adult pigs of 24 h when undertaken on higher standard vehicles and with constant access to water. For each journey from the farm to the abattoir, members of the research team accompanied the truck to ensure and verify that the journey met the objectives of the study. All journeys always took the same route and had the same lorry and driver.

The vehicle that was used was an articulated lorry with a tractor unit (MAN, Munich, Germany) towing a trailer (Carrozzeria Pezzaioli, Montichiari, Italy). The trailer had three floors with six compartments per floor (each compartment measured 220 cm long × 245 cm wide and 84 cm high), giving a total surface area of 5.39 m^2^ per compartment and an average stocking density of 0.42 m^2^/pig). The total loading capacity of the truck was about 27,000 kg and was equipped with suitable drinking systems with nipples to provide water during the journey. The trailer had both natural and mechanical ventilation systems, which consisted of twelve automatic fans per floor or six per side. The fans, nine in each truck, were 225 mm in diameter with a 11,700 m^2^/h flow in compliance with EC 1/2005. The trailer had a hydraulic controlled elevator for loading and unloading and provided anti-slip floors with incorporated side guards.

### 2.2. Enthalpy Assessment

Data on temperature and relative humidity were collected inside the livestock vehicles during loading, transport, and unloading using Hobo data loggers (Hobo H8 loggers, Onset Computers, Bourne, MA, USA). Prior to loading, two loggers were placed on the lorry at the same level as the pigs in the middle floor of the trailer, and the loggers had an inside that was specifically designed perforated metal tubing to let air in while avoiding contact with the animals. Sensors were pre-programmed to record temperature and relative humidity at regular 5 min intervals and were fitted and removed by a member of the research team before and after each journey. Approximately 20 mm of wood shavings were placed on each floor of the vehicle as bedding. 

### 2.3. Slaughter

The abattoir operated from Monday to Friday (from 06:00 a.m.to 15:00 p.m.) with a slaughter capacity of 2000 head/day at a rate of 220 heads/h. On arrival at the abattoir, pigs were unloaded with an adjustable-slope metal ramp with an anti-skid floor. After unloading at the abattoir, the pigs were showered for 15 in the winter or for 30 min in the summer, and the pigs were kept in lairage pens without mixing the groups on arrival and were given access to water through nipple drinkers. The lairage time for all animals was at least 12 h from arrival, including overnight rest and slaughter the following morning. At the end of the lairage period, pigs were stunned using a CO_2_ chamber with 70% CO_2_ atmosphere for approximately 60 s in a one-gondola dip-lift system. Following stunning, pigs were horizontally exsanguinated. Carcasses were then eviscerated and split before being placed in a chiller set at 4 °C for 24 h.

### 2.4. Physiological Assessment

Blood samples were taken at the time of slaughter to evaluate physiological stress from 20 pigs per journey (one 10 mL tube per animal, with anticoagulant, EDTA-K3), totaling 160 sampled animals. Once all of the samples were collected for each journey, the samples were refrigerated for 10 h until they were centrifuged at 1300× *g* for 10 min to obtain plasma. The parameters measured in the plasma were cortisol, glucose, and creatine kinase. Plasma cortisol was assessed by ELISA. Calibrators were prepared with vials of cortisol in PBS and BSA and lyophilized at the concentrations of 0, 10, 30, 100, 300, and 900 ng/mL. For the colorimetric reading of samples, the blank and calibrators were performed within 20 min from the end of the assay using a spectrophotometer (Hitachi 717^®^) at 405 (for concentrations below 30 ng/mL) and 450 nm (for concentrations between 30 and 900 ng/mL). Plasma glucose was determined by the enzymatic colorimetric method (GOD/PAP). All of the solutions were pipetted into a cuvette and were incubated for 20 min at room temperature (15–25 °C). The absorbance of the samples and the standard absorbency was read against the blank using a spectrophotometer (Hitachi 717^®^) at 505 nm. CK levels were measured using a Roche/Hitachi 717 Chemistry Analyzer (Roche Diagnostics, S.L., Sant Cugat del Valles, Spain) with Boehringer Mannheim reagents. 

### 2.5. pH Measurements

Muscle pH was measured on the carcasses from the 160 animals sampled during the slaughter. After slaughter and dressing but before carcass cooling the initial pH of the loin was measured (pH 45 min) using a portable pH meter (HANNA, mod. HI9125) with temperature compensation. The electrode was inserted in the *Longissimus dorsi* muscle (LD) between the 13th and 14th intercostal space, perpendicular to the midline of the left held carcass, at an average depth of 2.5 cm. Afterward, the ultimate pH was measured from the same animals at 24 h post-mortem at the same location on the carcass. 

### 2.6. Enthalpy Models and Statistical Analyzes

Psychrometric graphs were obtained for each of the eight journeys using data collected by the sensors placed at animal height inside the vehicles. The graphs were obtained based on the ASBE model, which included temperature, relative humidity, absolute humidity and enthalpy. The psychrometric data ASAE D271.2, defined in April 1979 and reviewed in 2005 (ASABE 2006, ST. Joseph, MI, USA), were used to calculate the psychrometric properties of the air surrounding the pigs. The temperature gradient was calculated using the Savitzky–Golay algorithm for one dimension, tabulating the data. The numeric derivatives were calculated using the Savgol routine in Matlab version 7.0 (Mathworks Inc., Natick, MA, USA). A polynomial routine was also used to test the neighboring data around each point. The points were processed by replacing them with the value of the polynomial. The derivatives were presented after programming the derivatives of the polynomials of each point. A window of 21 points with a fifth order polynomial was used. After that, we calculated and graphed the speed of temperature change (i.e., temperature gradient °C/s) and the apparent temperature in each case [19,20].Enthalpy (h) is a thermal comfort index that expresses the heat amount in 1 kg dry air in kJ and is determined by the equation as seen in Barbosa-Fihlo et al. [21].

H=(6.7+0.243t+((RH100)·10[7.5t237.3+t]))4.18

where:

H = enthalpy (kJ/kg dry air);
t = temperature (°C);
RH = relative humidity (%).

All of the statistical analyses were performed using the statistical program SAS/STAT (Statistical System Institute Inc. Cary, NC, USA. 2000). The data for temperature and humidity were analyzed using repeated measures, while the data on cortisol, glucose levels, and meat pH were analyzed using PROC MIXED. The experimental unit was each journey. Averages were compared by the least significant distance, with a level of significance of 5% (*p* < 0.05).

## 3. Results

**Figure 1:**
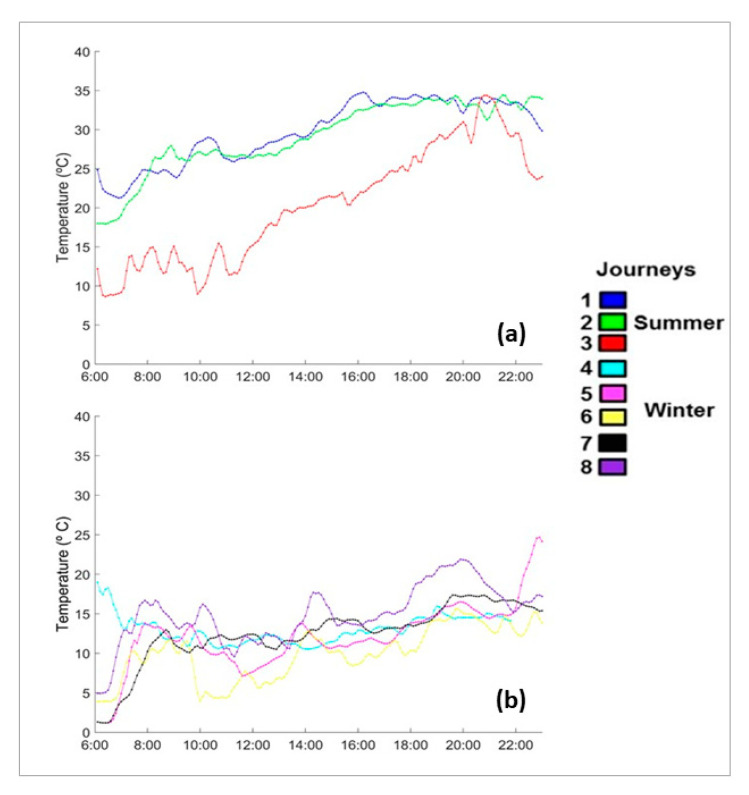
Evolution of the average temperatures (in °C) inside the livestock vehicle during transport from Spain to Portugal in (**a**) summer and (**b**) winter throughout the day beginning from loading at 6:00 until unloading at 23:00.

**Figure 2:**
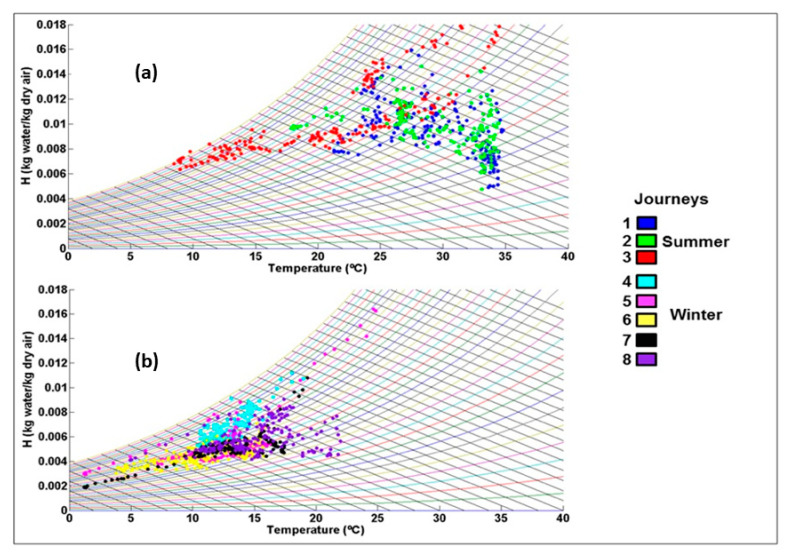
Psychrometric graph of the average temperatures (in °C) inside the livestock vehicle during transport from Spain to Portugal in (**a**) summer and (**b**) winter throughout the day with humidity (H kg water/k of dry air) plotted against temperature for each journey.

**Figure 3:**
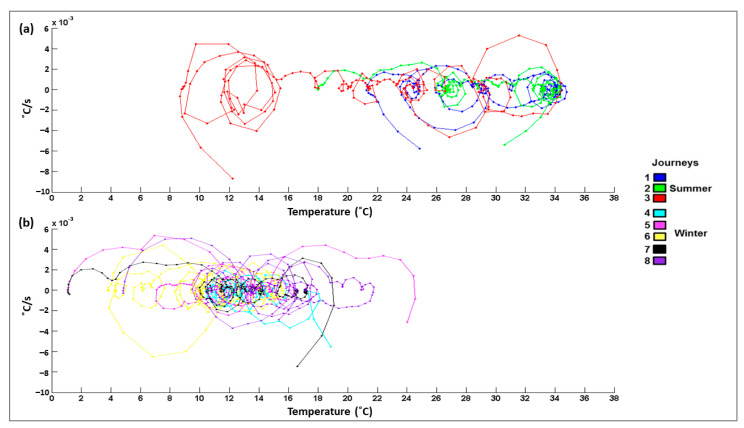
Speed of change or gradient in temperature (°C/s) inside the livestock vehicle during transport from Spain to Portugal in (**a**) summer and (**b**) winter plotted against the average temperatures (°C).

In the eight journeys that were studied, there was no mortality or non-ambulatory animals. There were five journeys that corresponded to the winter season (January–February), and the remaining three occurred in the summer season (June–August). Table 1 summarizes the average temperatures and relative humidity for each journey. The average inside temperature during winter journeys was 13.8 ± 3.9 °C (CV = 28.3%) and was 28.9 ± 4.1 °C (CV = 14.0%) in the summer. Figure 1 shows the changes in temperature inside of the truck over time for both the summer and winter journeys. During most journeys, the temperature increased as the transport progressed, with two journeys in summer surpassing 30 °C. Figure 2 shows the psychrometric graph for each journey, with more data points for the summer journeys in the upper right quadrant (higher temperatures and higher water content in the air). The average humidity for the winter months was 5.5 g water/kg dry air and was 9.8 g water/kg dry air in the summer. Figure 3 summarizes the speed of temperature change or gradient for the summer and winter journeys. The average speed of change for the winter months was 11.8 H/min^−1^ and for the summer months, it was 12.8 H/min^−1^. On average, plasma cortisol levels (±SD) were significantly higher (*p* < 0.05) in the winter (57.8 ± 11.7 nmol/L) than in the summer (28.8 ± 4.3 nmol/L). Plasma glucose was also significantly higher (*p* < 0.05) in the winter (309.7 ± 37.9) than in the summer (60.2 ± 26.6). CK exhibited no significant differences among the seasons (*p* > 0.05) and averaged 4887.3 ± 2649.1 U/L. The pH (±SD) of the LD muscle after 45 min was significantly lower (*p* < 0.05) in the winter (6.08 ± 0.24, range 5.29–6.56) compared to in the summer (6.24 ± 0.24, range 5.33–6.8). However, the pH after 24 h was not significantly different between the seasons (*p* > 0.05) and was 5.56 ± 0.13 (range 5.34–5.94).

## 4. Discussion

Since the beginning of the 20th century, pig production has continued to undergo massive intensification and specialization in most industrialized countries, leading to larger and fewer farms and abattoirs with increased distances between them [22]. Long-distance transport has been reported to be physically, metabolically, and emotionally very demanding for animals [23]. Thermal stress is one of the key factors that can exacerbate the effects of long-distance transport on pig health and welfare [24]. In this context, the current study shows that winter transport is thermally more unstable, with abrupt changes in temperature and enthalpy compared to during the summer. These microclimatic conditions have a clear impact on animal-based welfare indicators such as cortisol and glucose as well as on pH after 45 min, but this difference disappears between the seasons after 24 h. Overall, the results suggest that enthalpy during transport can be a useful non-invasive indicator of animal welfare.

Thermal stress is defined by the inability to maintain a constant body temperature by behavioral and physiological adaptation alone. This inability can result in heat stress or cold stress and, in extreme or prolonged cases, welfare consequences can lead to multi-organ failure and death [25]. The European Commission [16], has established a maximum temperature at which livestock, including pigs, must be transported on long journeys (i.e., 35 °C), but there are no accompanying limits for relative or absolute humidity. The thermoneutral zone of 100 kg pigs is centered around 20 °C [26], but the results from the current study show that temperature variations during transport vary much more widely, from about 5 to 35 °C. The coefficients of variation and gradients in the relative humidity and enthalpy were also high, especially in the winter, suggesting sudden shifts in the thermal environment around the animals. The results from the physiological parameters and the meat quality measurements suggest that pigs subjected to sharper enthalpy gradients had poorer welfare. 

The enthalpy ranges reported here are similar to a previous study by the research group that analyzed long-distance pig transport from the UK to Spain [15]. The psychrometric graphs underline the large differences between winter and summer journeys, with higher temperatures and lower humidity in the former and the opposite in the winter, as also seen in Seedorf et al. [27] and Lucas et al. [9]. Psychrometric graphs are not commonly used to model the microenvironment around livestock during transport although they can be used to calculate the absolute humidity and thus provide a better idea of the effort that animals need to make to lose heat to the environment. Indeed, the THI index can be graphed onto the psychrometric graphs themselves to designate danger zones. Using GPS data, we could then backtrack and find where along the way the thermal microenvironment may be more challenging on-board. 

Extreme ambient temperatures during live transport are considered to be one of the most relevant risk factors for injuries (both ambulatory and non-ambulatory) and deaths on arrival rates, especially as transport generally occurs when microclimate conditions are outside the ideal thermal comfort zone of the pigs [28]. However, the results presented here suggest that even though there was no mortality among the 1920 pigs that were transported, some winter journeys with high variation in temperature may be pushing the coping abilities of the animals. Other authors [29] have observed marked mortality and carcass defects in pigs transported in the winter compared to those transported in the summer in Spain.

During long-distance transport pigs are exposed to stressful events such as a new environments, new smells and noises, loading and unloading, mixing with unknown animals, deprivation of food, among others factors [30]. Elevation of plasma cortisol and glucose concentrations are considered to be more sensitive and reliable indicators to reflect the intensity of the stress response during transport [31]. The results showed that the animal-based measurements of the stress response, cortisol and glucose, showed significant differences between the seasons, with the winter being more stressful than the summer. These physiological measures validate the results concerning enthalpy rates. However, for the plasma CK enzyme variable, no significant differences were found between the summer and winter journeys. Although the average values we found for this enzyme are three times higher than those reported in pigs of a similar category during journeys of less than one hour in the Iberian Peninsula reported by Oliván et al. [32], CK is an important biochemical marker used to measure muscle exhaustion and fatigue during pig transport [33,34] because of the greater the amount of muscle microtrauma and the greater passage of this enzyme to the extracellular environment [7]. It is possible that the high CK levels found are the result of the interaction between thermal and environmental conditions (sensory stimuli, social interactions, density), the duration of the journey, and lairage time.

Acute thermal stress immediately before slaughter accelerates muscle glycogenolysis, increases lactic acid concentration, and produces a rapid decrease in muscle pH early post-mortem while the carcass is still hot [35]. In pigs, this results in pale, soft, and exudative (PSE) meat characterized by a lower water holding capacity. In contrast, animals subjected to chronic heat stress have reduced muscle glycogen reserves, leading to lower production of lactic acid and dark, firm, and dry (DFD) meat characterized by high ultimate pH and greater water holding capacity [36]. The results show that the pH after 45 min was affected in the pigs that were transported in the winter, however the average values remained within normal ranges. Surprisingly, at 24 h, this effect disappeared, which possibly related to the good conditions of the vehicles and the logistics implemented for these types of journeys. This may be due to the fact that European Legislation provides a series of guidelines that have substantially improved animal transport in the region [18]. It is possible that the same type of journey, without forced ventilation, could have greater heat stress effects on product quality [37].

## 5. Conclusions

Our results correspond to journeys under commercial conditions in specialized trucks, with a modern pre-slaughter logistics chain and following European regulations governing pork transport. Under these conditions, long-distance journeys during the winter in the Iberian Peninsula presented abrupt variations in the rates of temperature change and air enthalpy. These abrupt changes were reflected in higher values of cortisol and glucose, but not in CK. Muscle pH was affected at 45 min although at 24 h, these effects were not observed. Our study has shown that mitigation strategies to avoid thermal stress should be aimed at controlling abrupt changes in rates of temperature change and air enthalpy during long-distance transport.

## Figures and Tables

**Table 1:** Average temperature (T) and relative humidity (RH) inside (pig level) and outside the livestock vehicle during each pig transport journey from Spain to Portugal.

Journey	Season	T_int_ (°C)	T_ext_ (°C)	RH_int_ (%)	RH_ext_ (%)
1	Summer	20.6	24.3	66.9	52.3
2	Summer	29.2	29.3	38.2	38.8
3	Summer	29.7	32.9	34.7	37.7
4	Winter	13.7	15.1	76.9	72.5
5	Winter	16.4	15.2	56.9	57.2
6	Winter	11.6	6.8	36.4	57.5
7	Winter	12.6	9.7	52.9	56.9
8	Winter	14.6	19.8	52.6	44.7

T_int_: inside temperature; T_ext_: exterior temperature; RH_int_: inside relative humidity; HR_ext_: exterior relative humidity.

## Data Availability

The raw data have not been published or stored elsewhere but are available upon request.
